# The Relationship between Components of Postural Control and Locomotive Syndrome in Older Adults

**DOI:** 10.3390/ijerph21101349

**Published:** 2024-10-11

**Authors:** Charupa Lektip, Chadapa Rungruangbaiyok, Jiraphat Nawarat, Eiji Miyake, Keiichiro Aoki, Hiroyuki Ohtsuka, Yasuko Inaba, Yoshinori Kagaya, Sirawee Chaovalit

**Affiliations:** 1 Department of Physical Therapy, School of Allied Health Sciences, Walailak University, Nakhon Si Thammarat 80160, Thailand; charupa.le@wu.ac.th (C.L.); chadapa.bn@wu.ac.th (C.R.); nsuparoe@wu.ac.th (J.N.); 2 Movement Sciences and Exercise Research Center, Walailak University, Nakhon Si Thammarat 80160, Thailand; 3 Department of Physical Therapy, School of Nursing and Rehabilitation Sciences, Showa University, Tokyo 142-6555, Japan; e.miyake@nr.showa-u.ac.jp (E.M.); k.a-0525@cmed.showa-u.ac.jp (K.A.); ohtsuka@nr.showa-u.ac.jp (H.O.); inaba@nr.showa-u.ac.jp (Y.I.); kagaya@nr.showa-u.ac.jp (Y.K.); 4 Department of Physical Therapy, Faculty of Medicine, Prince of Songkla University, Songkhla 90110, Thailand

**Keywords:** postural balance, locomotive syndrome, aged, gait disorders, accidental falls

## Abstract

Locomotive Syndrome (LS), a condition related to impaired mobility, is influenced by balance control, which comprises six components. Deficiencies in these components can lead to reduced mobility and decreased quality of life. This study aimed to evaluate the relationship between the components of postural control and LS in older adults using the Brief-BESTest. Therefore, this cross-sectional study involved 122 elderly participants from Tha Sala District, Nakhon Si Thammarat Province, both with and without LS. Participants underwent assessments using the Instrumental Activities of Daily Living (IADL) assessment, the Thai Mental State Examination (TMSE), the Two-Step Test, and the Brief-BESTest. The Brief-BESTest covers six balance components: Biomechanical Constraints, Stability Limits, Anticipatory Postural Adjustments, Postural Responses, Sensory Orientation, and Stability in Gait. Descriptive statistics were used to summarize participant characteristics, and Chi-square tests were conducted to examine the relationship between each balance component and LS. Cramer’s V was used to assess the strength of the relationships. The results showed the average age of the sample was 67.67 ± 6.01 years with 85.20 percent female and 14.80 percent male. There were significant relationships between LS and three balance components: Biomechanical Constraints (Chi-square = 5.35, *p* = 0.021, Cramer’s V = 0.209), Stability Limits (Chi-square = 5.00, *p* = 0.025, Cramer’s V = 0.204), and Anticipatory Postural Adjustments (left: Chi-square = 4.12, *p* = 0.042, Cramer’s V = 0.213; right: Chi-square = 5.50, *p* = 0.019, Cramer’s V = 0.213). No significant associations were found for Reactive Postural Response, Sensory Orientation, and Stability in Gait. These findings suggest that targeted interventions focusing on specific balance components consist of Biomechanical Constraints, Stability Limits, and Anticipatory Postural Adjustments could help reduce the risk of LS in older adults.

## 1. Introduction

In the year 2022, Thailand experienced an increase in its elderly population, with those aged 60 and above accounting for 19.21% of the total population [1]. This group is comprised of 44.27% males and 55.73% females. Surveys indicate that falls among the elderly are a significant public health issue [2]. Especially during the COVID-19 outbreak, in the elderly, the ability to maintain balance decreases. This may be due to decreased participation in exercise [3]. The risk factors for falls include both internal factors (such as physiological adaptations in the elderly, including neurological and visual adjustments) and external factors (such as poor environmental conditions like slippery floors). These factors affect the mobility performance of the elderly, leading to abnormal movements. In addition, balance is affected by cognitive, motor, and physiological factors as well [4].

Currently, studies have identified a set of symptoms related to impaired movement known as “Locomotive Syndrome”, which is a condition characterized by impaired mobility due to the degeneration of bones, joints, and muscles, leading to difficulties in daily activities and an increased risk of needing care [5]. One of the factors influencing movements is balance control, which comprises seven components. A deficiency in at least one of these components can result in slower, less agile movement in the elderly, leading to limitations in daily life activities and a decrease in both physical and mental quality of life [6]. Research by Naveeda Ashraf et al. (2021) indicates that individuals with balance disorders or gait ataxia experience reduced movement, which is a risk factor for falls [7]. Additionally, research by Muramoto et al. (2012) supports that balance significantly affects Locomotive Syndrome. Their findings reveal a significant correlation between the Timed Up and Go (TUG) test and the GLFS-25 score, a measure used to stage Locomotive Syndrome [8].

Several recent studies have utilized the Berg Balance Scale (BBS), the Timed Up and Go test (TUG), and the Mini BESTest. However, both the BBS and TUG are functional balance tests that do not assess balance improvement in response to unforeseen disturbances, which is a crucial component of balance and essential in preventing falls among the elderly [9]. The BESTest (Balance Evaluation Systems Test) is an internationally standardized assessment tool that evaluates balance across six systems: (1) Biomechanical Constraints, (2) Stability Limits/Verticality, (3) Anticipatory Postural Adjustments, (4) Postural Responses, (5) Sensory Orientation, and (6) Stability in Gait. Research by Fay B. Horak et al. (2009) indicates that the BESTest is easy to study and has high reliability and validity. Moreover, the BESTest can specifically identify and treat balance problems in patients [10].

The BESTest assessment consists of 27 items, making it time-consuming to administer. Consequently, researchers have explored alternatives and found the Mini-BESTest, which evaluates overall balance components divided into four main areas: (1) Anticipatory, (2) Reactive Postural Control, (3) Sensory Orientation, and (4) Dynamic Gait. The Mini-BESTest has a total of 14 items, making it shorter and more concise, but it does not cover all six balance components, thus failing to comprehensively identify balance issues [11]. However, research by Noureddin et al. (2022) found that the internal and inter-rater reliability of the Mini-BESTest and the Brief-BESTest were excellent, with good correlations between these assessments and the BBS [12]. Brief-BESTest consists of eight items, covers all six balance systems, and is quick to administer. Furthermore, a study by Meizhen Huang et al. (2017) evaluated balance in stroke patients using the Brief-BESTest, concluding that it demonstrated good internal and inter-rater reliability [13].

Previous research on elderly individuals with Locomotive Syndrome has mainly focused on evaluating functional balance using tests like the Timed Up and Go (TUG) test. However, these studies have not provided a comprehensive assessment of all six components of balance control that contribute to movement impairments in the elderly. Furthermore, no studies have used the Brief-BESTest to assess balance control specifically in this population. Therefore, this study aims to fill these gaps by evaluating balance in older adults with Locomotive Syndrome using the Brief-BESTest and investigating the relationship between the six components of balance control and Locomotive Syndrome.

## 2. Method

### 2.1. Sample

This cross-sectional study examines a study population of elderly Thai males and females aged 60 and above, residing in the Tha Sala Subdistrict Municipality, Tha Sala District, Nakhon Si Thammarat Province, comprising 327 individuals [14], and in the Pho Thong Community, Pho Thong Subdistrict, Tha Sala District, Nakhon Si Thammarat Province, comprising 118 individuals [14], for a total population of 445 individuals.

The sample consists of 122 elderly males and females aged 60 and above (mean ± SD: 67.67 ± 6.01) who consented to participate in the research project. This includes elderly individuals both with and without Locomotive Syndrome. The sample size was calculated using the G*power program type chi-square (X^2^) statistical test, with a power of test set at 0.80, an effect size of 0.3, Degree of freedom (df) set at 3 and the significance level set at 0.05.

The sample was selected using purposive sampling based on the following inclusion criteria: (1) Elderly males and females aged 60 years and above. (2) Able to read, write, understand, and communicate in Thai. The exclusion criteria were as follows: (1) Presence of chronic diseases affecting balance and movement, such as stroke or Parkinson’s disease. (2) Experiencing pain in the hip, knee, or ankle joints on the assessment day. (3) Having disabilities, including hearing and speech impairments. (4) Experiencing dizziness on the test day. (5) Consuming alcoholic beverages within 24 h before the test. (6) Elderly individuals scoring 23 or less out of 30 on the Thai Mental State Examination (TMSE) [15]. (7) Elderly individuals scoring 3–4 (for those without a telephone, full score of 10) or 3–5 (for those with a telephone, full score of 12) on the Instrumental Activities of Daily Living (IADL) test, indicating severe dependence [16].

### 2.2. Instruments

The Instrumental Activities of Daily Living (IADL) assessment measures the ability of elderly individuals to perform various activities. It involves asking about daily activities the elderly have carried out within the past 24–48 h, aiming to assess their level of independence. If the elderly person has a caregiver or helper while performing daily activities, they do not receive full marks. The scoring is divided into two parts. For those without a telephone (maximum score of 10 points): Scores between 0 and 2 points indicate complete dependence. Scores between 3 and 4 points indicate severe dependence. Scores between 5 and 6 points indicate moderate dependence. Scores of 7 points and above indicate no dependence.

For those with a telephone (maximum score of 12 points): Scores between 0 and 2 points indicate complete dependence. Scores between 3 and 5 points indicate severe dependence. Scores between 6 and 8 points indicate moderate dependence. Scores of 9 points and above indicate no dependence. The IADL questionnaire has a Cronbach’s alpha coefficient of 0.32 (95% CI; −0.12, 0.66) [17].

The Thai Mental State Examination (TMSE) is a brief assessment used to evaluate cognitive function in elderly individuals, specifically to screen for dementia. It consists of six major questions, with a total score of 30 points. If an elderly person scores 23 points or fewer, it indicates the presence of dementia. The TMSE has a sensitivity of 75.52%, a specificity of 88.80% [18], and an internal consistency reliability of 0.82 [19].

The Two-Step Test is a simple and commonly used test to assess impaired mobility (Locomotive Syndrome) [20] and is used to differentiate between elderly individuals with impaired mobility (Non-Locomotive Syndrome) and those without impaired mobility (Locomotive Syndrome). The test involves having the elderly person stand on a line, then take two steps forward as far as possible. The distance covered is measured, and the test result is calculated using the formula: the length of the two steps (in centimeters) divided by the height of the elderly person (in centimeters) equals the step test score [21]. The risk levels for Locomotive Syndrome are categorized as follows: Level 1 risk: A step test score less than 1.3 indicates the elderly person is beginning to experience impairments in movement, strength, and muscle balance. Level 2 risk: A step test score less than 1.1 indicates advanced impairment in movement ability, with a high risk of limitations in daily activities [22]. The Two-Step Test has an Intraclass Correlation Coefficient of 0.93 and a Cronbach’s Alpha Coefficient of 0.95 [23]. 

The Brief-BESTest is used to evaluate balance and consists of eight items covering six components. Each item is scored from 0 to 3, with higher scores indicating better balance performance. The total score ranges from 0 to 24 [24]. The cutoff score for the Brief-BESTest, tested on elderly individuals in 15 communities in Japan, indicates that those at risk of falling have a cutoff score of 12.5 [25]. In tests conducted on patients with Parkinson’s disease, those with a history of falls in the past six months had a cutoff score of 11 [26]. The Brief-BESTest has a sensitivity of 67% and a specificity of 71%. The inter-rater reliability is 0.99 [27], and the Cronbach’s alpha coefficient is 0.85 [28].

### 2.3. Procedure

This study involved a structured process for selecting participants, screening for Locomotive Syndrome, and assessing balance control through various tools:

(1) Participant Selection:

The researcher began by administering a questionnaire to elderly individuals aged 60 and above living in the targeted community. The purpose of the questionnaire was to collect baseline demographic data and identify eligible participants for this study based on the inclusion and exclusion criteria.

(2) Screening for Daily Living and Cognitive Function:

The Instrumental Activities of Daily Living (IADL) Assessment was used to evaluate the participants’ independence in daily activities. Scores were calculated to categorize the level of dependence, ranging from complete independence to severe dependence.

The Thai Mental State Examination (TMSE) was administered to assess cognitive function. Participants with scores below the cutoff were excluded from further testing to ensure cognitive impairments did not confound the study’s outcomes.

(3) Assessment of Mobility:

The Two-Step Test was conducted to distinguish participants with and without Locomotive Syndrome (LS).

(4) Balance Assessment:

All participants completed the eight-item Brief-BESTest, which evaluates balance across multiple components, including biomechanical constraints, stability limits, anticipatory postural adjustments, and sensory orientation. 

All procedures performed in studies involving human participants were in accordance with the ethical standards of the institutional research committee. Ethical clearance was obtained from the Institute Review Board Walailak University (IRB reference no. WUEC-24-104-01).

### 2.4. Statistical Treatment

The statistical analysis of the data was carried out using the IBM SPSS Statistics v. 25.0. (IBM, Armonk, NY, USA). The analysis details are as follows: (1) Descriptive statistics were used to analyze the characteristics of the sample group. (2) The Chi-square test was used to find the relationship between each component of balance control and Locomotive Syndrome. The data were categorized with nominal variables (elderly individuals with and without Locomotive Syndrome) and ordinal variables (scores ranging from 0 to 3 on the Brief-BESTest). Cramer’s V statistic was used, with a significance level set at 0.05 (*p* < 0.05), to analyze the strength of the relationships. If the expected value is less than 5 for more than 20% of the cases, Fisher’s Exact Test (a non-parametric statistic) will be used to determine the relationship between each component of balance control and Locomotive Syndrome.

## 3. Results

The objective of this research is to investigate the relationship between balance components and mobility impairments in the elderly. The researcher summarized the study findings as follows: (1) Baseline characteristics of the sample group. (2) Results from the Two-Step Test and the total scores of the Brief-BESTest. (3) The relationship between balance control components and mobility impairments.

Baseline Characteristics of the sample group included a total of 122 participants, all elderly individuals residing in Tha Sala District. The majority of the participants were female and belonged to the early-to-mid elderly age group. Further details and characteristics are presented in Table 1.

**Figure 1:**
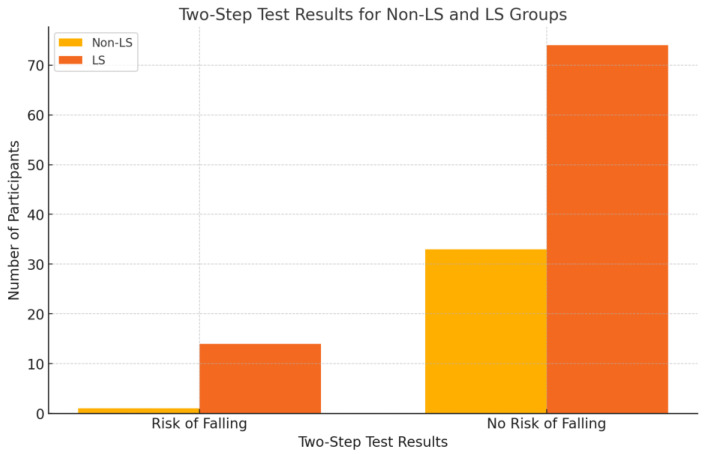
The total results of the Two Step Test and the overall scores of the Brief-BESTest evaluation.

As for the results from the Two Step Test and the total score of the Brief-BESTest balance assessment, this study found that, out of 122 volunteers, the Non-LS (No Locomotive Syndrome) group had only one participant at risk of falling, 33 participants with no risk of falling. In addition, the LS (Locomotive Syndrome) group had 14 participants at risk of falling, 74 participants with no risk of falling, as shown in Figure 1. The average total score from the Brief-BESTest was 17.07 ± 3.99 points, indicating a risk of falling. The cutoff score was set at 12.5 points [25].

The relationship between different components of balance control and movement impairments in the elderly is presented in Table 2. Significant associations were observed in four components, biomechanical constraints (Sig. = 0.021, Phi = 0.209), stability limits (Sig. = 0.025, Phi = 0.204), and transitions-anticipatory postural adjustment on both the left (Sig. = 0.042, Phi = 0.184) and right sides (Sig. = 0.019, Phi = 0.213), all interpreted as “High” associations. In contrast, reactive postural response (left: Sig. = 0.874, Phi = 0.014; right: Sig. = 0.539, Phi = 0.056) showed “Very low” and “Low” associations, respectively. Sensory orientation (Sig. = 0.116, Phi = 0.142) and stability in gait (Sig. = 0.188, Phi = 0.119) were found to have “Moderate” associations with movement impairments, where stability in gait had average time spent in the LS group = 7.52 ± 2.41 s and the non-LS group = 10.06 ± 2.69 s.

## 4. Discussion

In this study, our proposed objective was to study the possible relationship between the six components of balance and movement impairments (Locomotive Syndrome) in the elderly using the Brief-BESTest evaluation.

According to the research findings, component 1: Biomechanical constraints shows a statistically significant relationship with movement impairments (Locomotive Syndrome) with *p* = 0.021 and a high correlation coefficient Phi = 0.209. This aligns with the research by Kota Kato et al. (2020), which stated that aging or prolonged muscle inactivity results in decreased strength of the hip and trunk muscles [29,30]. Additionally, it aligns with the research by Ryosuke Tokida et al. (2020), who studied the relationship between the deterioration of bone and muscle function and movement impairments (Locomotive Syndrome) in the general elderly population. Their tests on knee extension strength and one-leg standing between elderly individuals with and without movement impairments (Locomotive Syndrome and Non-Locomotive Syndrome) found that knee extension strength had a significant relationship with movement impairments (Locomotive Syndrome) with *p* < 0.01, and one-leg standing had a significant relationship with movement impairments (Locomotive Syndrome) with *p* < 0.05 [31]. Furthermore, research by Simone Gafner et al. (2017) indicated that aging impacts hip muscle strength, which affects the risk of falling [30].

Based on the research findings in component 2: Stability limits, which involves the functional reach forward test, there is a statistically significant relationship with *p* = 0.025 and a high correlation coefficient Phi = 0.204. This finding is consistent with the research by Saithida Lapanunthasin et al. (2015), who studied elderly individuals with a fear of falling. When the elderly individuals participated in an exercise program to improve balance, the evaluation of their balance ability and fear of falling showed a statistically significant improvement in balance ability assessed by the Functional Reach Test (*p* = 0.05). This indicates that after the elderly individuals improved their balance ability, they could perform the Functional Reach Test better [32]. Additionally, research by Kathryn M. Sibley. et al. (2015) analyzed balance components using various standard evaluations and found that Functional Stability Limits are a primary component for balance assessment. This suggests that stability limits are likely a key component of balance that can indicate movement impairments (Locomotive Syndrome) [33].

Based on the research findings in component 3: Transitions-Anticipatory postural adjustment, there is a statistically significant relationship for both the left and right legs with *p* = 0.042 and *p* = 0.019, respectively, and high correlation levels at *p* = 0.184 and *p* = 0.213 for the left and right legs, respectively. This correlates with the research by Tanaka et al. (2019), which stated that the body’s center of gravity, when shifted forward or backward, increases the relationship with maintaining balance to control stability in individuals with movement impairments (Locomotive Syndrome) [34]. Additionally, research by Yuki Nishi et al. (2022) studied anticipatory postural control in elderly individuals with chronic lower back pain using a kinesiophobia assessment and lumbar movement test to observe the position of the center of pressure (COP) and its relationship with body position changes. The study concluded that anticipatory postural control adjustments in elderly individuals with chronic lower back pain affect kinesiophobia [35]. This is consistent with the research in question, as the test in component 3 involves lifting one leg backward, causing a change in the COP position and requiring the body to adjust to maintain balance.

In accordance with the research findings in component 4: Reactive Postural Response, there is no statistically significant relationship in the elderly with and without movement impairments (Locomotive Syndrome). The left side had a *p*-value of 0.874 and a Phi value of 0.014, which is very low. The right side had a *p*-value of 0.539 and a Phi value of 0.056, also low. According to the research by Ilaria Mileti et al. (2019), the ability of elderly individuals to respond with Reactive Postural Responses is reduced compared to younger individuals. The abnormal Reactive Postural Responses observed in the elderly may lead to difficulties in managing body balance control, potentially coupled with a fear of falling during testing in both elderly groups with and without Locomotive Syndrome [36]. This results in both groups being unable to adequately perform the Reactive Postural Responses test, hence, it does not show a significant relationship between Reactive Postural Response and movement impairments (Locomotive Syndrome).

According to the research findings in component 5: Sensory Orientation, there is no statistically significant relationship (*p*-value = 0.116) and a Phi value of 0.142, which is moderate. The research by Muyinat Y. Osoba et al. (2019) indicated that aging leads to a decline in the sensory system [37]. Similarly, research by Qipeng Song et al. (2021) stated that proprioception, which is one of the three inputs of the individual sensory system, decreases in sensitivity with age. This affects both dynamic and static balance control, impacting the ability to adapt to environmental changes and maintain balance [38]. Therefore, these issues may occur in all elderly individuals, which means that the relationship between Sensory Orientation and movement impairments (Locomotive Syndrome) cannot be demonstrated.

Based on the research findings in component 6: Stability in gait, there is no statistically significant relationship with elderly individuals who have and do not have movement impairments (Locomotive Syndrome) (*p*-value = 0.188), and the correlation coefficient is moderate at Phi = 0.119. This is because the TUG (Timed Up and Go) test scores are within a good range for both groups, indicating fast walking, a time of less than 11 s, and good balance for both elderly individuals with and without movement impairments (Locomotive Syndrome).

This study has limitations. The number of male participants was lower than female participants, which may limit the applicability of the results to elderly males. Moreover, different genders affect gait stability ratio (GSR) and body balance (BB) in the elderly as well [3]. Additionally, the research showed that the age range of the participants was in the early to middle elderly stages, and the sample group was from Thasala District, Nakhon Si Thammarat Province. Therefore, this study’s findings cannot be generalized to other regions, as populations in different areas may have varying occupations, lifestyles, and daily activities.

Therefore, future studies should collect data from an equal number of male and female participants, include late-elderly individuals, and use random sampling from different regions. This approach will ensure that the findings are more comprehensive and applicable to a wider population.

## 5. Conclusions

This study using the Brief-BESTest to find the relationship between balance components and movement impairments (Locomotive Syndrome) in the elderly found that three balance components are related to Locomotive Syndrome. These components are Biomechanical constraints (*p* = 0.021, Phi = 0.209), Stability Limits (*p* = 0.025, Phi = 0.204), and Transitions-Anticipatory postural adjustment on the left side (*p* = 0.042, Phi = 0.213) and the right side (*p* = 0.019, Phi = 0.213).

## Figures and Tables

**Table 1:** Baseline characteristics of the sample group (n = 122).

Baseline Characteristics		Non-LS (n = 34)	LS (n = 88)	*p*-Value
		n (%)	n (%)	
Age (years)				0.102 ^b^
Mean ± SD		65.72 ± 5.39	67.81 ± 6.38	
Gender	Male	2 (5.88)	16 (18.18)	0.086 ^c^
	Female	32 (94.12)	72 (81.82)	
BMI ^a^ (kg/cm^2^)				0.056 ^b^
Mean ± SD		24.96 ± 2.91	26.32 ± 4.65	
Religion	Buddhist	23 (67.65)	44 (50.00)	0.079 ^c^
	Muslim	11 (32.35)	44 (50.00)	
Marital Status	Single	3 (8.82)	9 (10.23)	0.474 ^c^
	Married	25 (73.53)	52 (59.09)	
	Widowed	5 (14.71)	24 (27.27)	
	Divorced	1 (2.94)	3 (3.41)	
Education Level	Primary	17 (50.00)	61 (69.32)	0.060 ^c^
	Secondary	6 (17.65)	19 (21.59)	
	Higher than Secondary	11 (32.35)	8 (9.09)	
Occupation	Laborer	11 (32.35)	33 (37.50)	0.097 ^c^
	Trader	5 (14.71)	20 (22.73)	
	Housewife	6 (17.65)	19 (21.59)	
	Own Business	3 (8.82)	1 (1.13)	
	Retired Government Official	8 (23.53)	9 (10.23)	
	Unemployed	1 (2.94)	6 (6.82)	
Income	No income	1 (2.94)	5 (5.68)	0.116 ^c^
	Less than 5000	15 (44.12)	56 (63.63)	
	5000–15,000	8 (23.53)	15 (17.05)	
	15,001–30,000	10 (29.41)	12 (13.64)	
History of falls in the past 6 months	Not falling	30 (88.24)	70 (79.54)	0.089 ^c^
	Falling	4 (11.76)	18 (20.46)	

^a^: Body mass index. ^b^: Independent sample *t*-test. ^c^: Chi-squared test.

**Table 2:** The relationship between the components of balance control and movement impairments.

Items	Sig.	Phi	Interpretation
Biomechanical constraints	0.021 *	0.209	High
Stability limits	0.025 *	0.204	High
Transitions-Anticipatory postural adjustment (Lt.)	0.042 *	0.184	High
Transitions-Anticipatory postural adjustment (Rt.)	0.019 *	0.213	High
Reactive postural response (Lt.)	0.874	0.014	Very low
Reactive postural response (Rt.)	0.539	0.056	Low
Sensory orientation	0.116	0.142	Moderate
Stability in gait	0.188	0.119	Moderate

The Pearson Chi-Square was performed. * Significant difference, *p* < 0.05.

## Data Availability

The data that support the findings of this study are available from the corresponding author upon reasonable request.
